# Early trauma and schizophrenia onset: preliminary results of an outpatient cohort in Brazil

**DOI:** 10.47626/2237-6089-2020-0024

**Published:** 2022-10-18

**Authors:** Leandro de Oliveira Trovão, Gilberto Sousa Alves, Carolina Gomes Carrilho, Thaysse Gomes Ricci, Lays Bittencourt, Cândida Alves, Natália Costa Brito, Antonio Egídio Nardi, Dolores Malaspina, André Barciela Veras

**Affiliations:** 1 Grupo de pesquisa em Psiquiatria Translacional Hospital Nina Rodrigues Universidade Federal do Maranhão São Luís MA Brazil Grupo de pesquisa em Psiquiatria Translacional , Hospital Nina Rodrigues , Universidade Federal do Maranhão , São Luís , MA , Brazil .; 2 Programa de Pós-graduação em Psiquiatria e Saúde Mental Universidade Federal do Rio de Janeiro Rio de Janeiro RJ Brazil Programa de Pós-graduação em Psiquiatria e Saúde Mental (PROPSAM), Universidade Federal do Rio de Janeiro (UFRJ), Rio de Janeiro , RJ , Brazil .; 3 Grupo de Pesquisa Translacional em Saúde Mental Universidade Católica Dom Bosco Campo Grande MS Brazil Grupo de Pesquisa Translacional em Saúde Mental (GPTranSMe), Universidade Católica Dom Bosco , Campo Grande , MS , Brazil .; 4 Laboratório de Neurociências Universidade Ceuma São Luís MA Brazil Laboratório de Neurociências , Universidade Ceuma , São Luís , MA , Brazil .; 5 Laboratório de Pânico e Respiração Instituto de Psiquiatria UFRJ Rio de Janeiro RJ Brazil Laboratório de Pânico e Respiração (LabPR-UFRJ), Instituto de Psiquiatria (IPUB), UFRJ , Rio de Janeiro , RJ , Brazil .; 6 Departments of Psychiatry, Neuroscience and Genetics Icahn School of Medicine Mt. Sinai Medical Center New York NY USA Departments of Psychiatry, Neuroscience and Genetics , Icahn School of Medicine , Mt. Sinai Medical Center , New York , NY , USA .

**Keywords:** Early trauma, schizophrenia, abuse, depression

## Abstract

**Objectives:**

To assess the prevalence of early trauma in individuals with onset of schizophrenia (SZ) at early (≤ 18 years) and adult (> 18 years) ages (EOP and AOP, respectively) and explore relationships between the onset of disease and clinical variables including traumatic events and psychotic and mood symptoms.

**Methods:**

Subjects with SZ (n = 71) and EOP and AOP were compared for history of psychological trauma, sexual abuse, and physical punishment using the Early Trauma Inventory Self Report - Short Form (ETISR-SF). They were also compared for history of comorbidities and affective disorders using the Diagnostic Interview for Psychosis and Affective Disorders, the Positive and Negative Syndrome Scale, the Liebowitz Social Anxiety Scale, and the Calgary Depression Scale for Schizophrenia. Coefficients were calculated for correlations between scale results and disease duration.

**Results:**

Early trauma was significantly associated with an early onset psychotic episode (r = -0.315, p < 0.01). General trauma and depressive symptoms in adulthood were also associated (r = 0.442, p < 0.01), as were social anxiety symptoms and early trauma (r = 0.319, p < 0.01). Total ETISR-SF scores and the physical abuse item were significantly higher in EOP than in AOP. In the hierarchical regression, PANSS scores were best predicted by a model including the duration of disease and age of first psychotic episode (R = 0.303).

**Conclusions:**

Our results support the hypothesis that early trauma, including physical abuse, may play a relevant role in schizophrenia symptoms, such as an earlier psychotic occurrence, as well as features of other psychiatric disorders, such as greater severity of social anxiety and depression.

## Introduction

Schizophrenia (SZ) is a severe psychiatric condition characterized by psychosis, a decline in function and deficits in social and emotional function, and cognitive impairments. ^1^ The worldwide prevalence of schizophrenia is estimated at 0.28 to 0.7%, with onset most typically occurring in late adolescence and mid-30s. ^2^ Although pathogenic mechanisms are unknown, an interaction between genetic, molecular, environmental, and neurodevelopmental factors seems to be relevant. ^1 - 4^

Advances in genetic research have shown that the most influential association with schizophrenia at a population level involves variants of the Major Histocompatibility Complex (MHC) locus, more specifically, in parts of various structurally diverse alleles of complement component 4 (c4) genes. ^4^ In this context, environmental exposure to psychological stress factors with early trauma can also increase the risk of phenotypic expression of the disease. ^5^ Emotionally traumatic experiences occurring in early life stages seem to influence the clinical features of schizophrenia, apparently promoting greater severity of cognitive deficits and productive symptoms, including hallucinations and delusions. ^6^ Deregulation of the hypothalamic-pituitary-adrenal axis (HPA) and hippocampal neuronal loss are commonly implicated mechanisms. Increased release of stress-related glucocorticoids is associated with hippocampal atrophy, resulting in learning and memory disabilities. Furthermore, a high percentage of individuals with SZ show heightened levels of basal cortisol or non-suppression of dexamethasone, supporting activation of the HPA axis. ^7^ Prenatal stress, maternal separation, and childhood abuse and emotional neglect are associated with the severity of both psychotic and depressive symptoms in adulthood and an overall reduction in brain volumes and increased amygdala compared to the whole brain volume. ^8^ Mood symptoms and anxiety also seem to be strongly influenced by traumatic experiences, with correlations with premorbid symptoms and comorbidities in schizophrenia.

Depressed mood, anhedonia, and social withdrawal, which are frequent in schizophrenia, ^9 , 10^ could be related to early trauma, as observed in post-traumatic stress disorder (PTSD). ^11^ Moreover, anxiety and depression might be influenced by the prevalence of early trauma in SZ, and may be related to neurobiological changes, such as HPA axis hyperactivity. ^11^ Early life experiences influence the development of relationship patterns in adulthood and might also do so among those with schizophrenia. ^10^

Another important topic is how early exposure to traumatic experience may influence the onset of SZ symptoms. There are several hypotheses associating premorbid factors with early onset of psychosis. Epidemiological studies have suggested that psychotic-like experiences are more common by the age of 12 in children previously exposed to childhood trauma. ^12^ Child and adolescent abuse and neglect seemed to result in vulnerability to development of positive and negative symptoms. ^13^ According to the epigenetic hypothesis, ^14^ experiencing trauma would trigger a set of neurobiological and gene-environment changes, leading to affective disturbances and emotional distress (anxiety, tension, depression) and ultimately to clinical psychosis. ^15^ In some studies, psychosis breakdown during adolescence (early-onset psychosis – [EOP] [≤ 18 years]) has been associated with a history of family violence and sexual abuse. ^16 , 17^ Studies have shown that onset in adulthood (adult-onset psychosis – [AOP] [> 18 years]) is less linked to social problems or a history of family violence. Furthermore, EOP patients were likely to have worse premorbid functioning and a significantly longer duration of untreated psychosis than those with later onset. ^18 , 19^

The overarching aim of this study was to assess the prevalence of early trauma in individuals with schizophrenia and examine the relationship between these events and symptoms in schizophrenia, including psychiatric comorbidities, and to test the hypothesis of whether early exposure to psychological or physical abuse was associated with younger age of onset and more severe clinical features.

## Method

The study selected 71 individuals previously diagnosed with schizophrenia according to the Diagnostic and Statistical Manual of Mental Health, 5th edition, (DSM-5) ^3^ and ICD-10 (International Classification of Diseases) criteria. ^20^ These patients were consecutively assessed from July 2017 to April 2019 at the Nina Rodrigues psychiatric hospital in São Luís, state of Maranhão, northern Brazil, during specialized outpatient care, after being discharged from the psychiatric ward. Patients were excluded if they had premorbid organic mental disorders (e.g., epilepsy, brain tumors, AIDS, other CNS infections, significant head trauma, or intellectual disability) or a significant premorbid history of addiction to illicit drugs or alcohol.

All subjects were interviewed alongside their parents or guardian to confirm and supplement medical history and assessment instruments. Clinical judgment was necessary for the response of the caregiver or patient to be considered relevant. In cases of psychosis or symptomatic exacerbation, the information provided by the caregiver prevailed. An experienced mental health professional assessed each patient before enrolment. In cases where patients did not understand a question, the questionnaire was redesigned and adapted for the patient’s level of cognition and cultural understanding. Finally, all patients’ official medical records were also reviewed. To ensure the accuracy of data, all participants’ diagnostic information was discussed and reviewed with the team by the research coordinators in Brazil (A.B.V. and G.S.A).

The study was approved by the National Research Ethics Committee (CAAE 45604215.3.1001.5162). The subjects were first informed about the research by the clinical staff and all subjects signed informed consent, with additional written authorization obtained from the subjects’ relatives, according to the terms of the Helsinki Declaration.

Clinical assessment was followed by administration of structured interviews, scales, and inventories. The Diagnostic Interview for Psychosis and Affective Disorders (DI-PAD), an interview-based on ICD-10 and DSM IV algorithms, ^21^ was used to assess schizophrenia-related symptoms (positive and negative symptoms), depression, and mania. Positive and negative symptoms were also assessed with a validated Brazilian Portuguese version of the Positive and Negative Syndrome Scale (PANSS). ^22 , 23^ Comorbidities related to anxiety disorders were assessed with the validated Brazilian Portuguese version of the Liebowitz Social Anxiety Scale (LSAS). ^24 , 25^ The Calgary Depression Scale, validated for use in Brazil, was used to investigate depressive symptoms for Schizophrenia. ^17^ The Early Trauma Inventory Self Report-Short Form (ETISR-SF) was used to assess the prevalence of traumata. This inventory assesses early trauma, that is, before the age of 18, according to the following categories: general trauma, and physical, sexual, and emotional abuse. Interviewees can answer yes or no to each of the 27 items assessed. This scale was translated to Portuguese, adapted to the Brazilian context and assessed for validity and reliability, presenting adequate indicators. ^10 , 26 , 27^

### Statistical analysis

Absolute and relative frequencies were used to describe qualitative variables and the minimum and maximum values, mean, standard deviation, and 50% percentile for quantitative variables. The Shapiro-Wilk test was used to evaluate the normality of variables and Spearman coefficients were calculated in R to assess correlations between ETISR-SF scale domains and other study variables. Group comparisons between EOP and AOP were performed using Student’s *t* test. The significance level adopted was 5% (p < 0.05) and Data Analysis and Statistical Software (STATA®) version 14.0 was used to tabulate and analyze data. Finally, a hierarchical regression model was constructed with sociodemographic and clinical variables entered in blocks (including traumata scores) as potential predictors, with total PANSS scores used as dependent variable.

## Results

Table 1 shows the main clinical characteristics of the participants. Most of the subjects were adults older than 30 years, with disease onset occurring earlier in life before the age of 19 ( Table 1 ). Descriptive statistics for the ETISR-SF, Calgary, Liebowitz, and PANSS scale scores are shown in Table 2 . The highest mean score observed for categories of traumata experienced before the age of 18 was for general trauma. There was considerable variation among patients in terms of the total number of ETISR-SF scale items endorsed. The mean and SD scores on the Calgary Depression Scale for schizophrenia were deemed low, ^28^ and subjects tended to exhibit low to middle scores on the Liebowitz scale for fear/anxiety and avoidance. ^24^ On the PANSS scale, negative symptoms, such as emotional withdrawal and lack of attention, were more frequent than positive symptoms ( Table 2 ).

**Table 1:** Sociodemographic and clinical characteristics of schizophrenic patients, São Luís, MA, Brazil, 2018 (n = 71)

Variables	n	%
Gender		
Female	22	30.99
Male	49	69.01
Age		
≤ 18 years	3	4.23
19 – 30 years	26	36.62
> 30 years	42	59.15
Minimum	16.00	
Maximum	62.00	
Mean (SD)	35.57(12.21)	
Percentile 50%	36.00	
Age of first psychotic episode		
≤ 18 years	33	46.48
19 - 30 years	28	39.44
> 30 years	10	14.08
Minimum	7.00	
Maximum	61.00	
Mean (SD)	21.80 (9.60)	
Percentile 50%	19.00	

SD = standard deviation.

**Table 2:** Descriptive statistics of the ETISR-SF, Calgary, Liebowitz, and PANSS scales scores for schizophrenic patients (n = 71)

Variables	Mean (SD)	Minimum	Maximum	Percentile 50%
ETISR-SF				
General trauma	3.13 (2.55)	0	10	3
Physical abuse	1.66 (1.69)	0	5	1
Emotional abuse	2.23 (1.90)	0	5	2
Sexual abuse	1.04 (1.78)	0	6	0
Reaction to trauma	0.77 (0.87)	0	4	1
Total	8.61 (6.35)	0	25	6.5
Calgary				
Total	5.01 (5.47)	0	24	4
Liebowitz				
Fear and anxiety	18.76 (16.88)	0	64	15
Avoidance	16.55 (16.93)	0	64	11.5
PANSS				
Positive scale	18.37 (8.09)	7	41	16
Negative scale	21.01 (9.91)	9	46	20
Total score	77.71 (26.48)	35	169	72

Calgary = Calgary Scale for Depression in Schizophrenia; ETISR-SF = Early Trauma Inventory Self Report-Short Form; Liebowitz = Liebowitz Social Anxiety Scale; PANSS = Positive and Negative Syndrome Scale; SD = standard deviation.

### Results for AOP and EOP subsets

Table 3 shows the group comparisons for disease onset. AOP individuals were significantly older than EOP participants, although they had presented the disease for fewer years. Total ETISR-SF scores and the physical abuse item were significantly higher among EOP than AOP. However, there were no statistical differences between the groups in Calgary or Liebowitz scale scores.

**Table 3:** Clinical comparisons of groups according to age at disease onset

	EOP (Mean-SD) n = 34	AOP (Mean-SD) n = 37	F (df)	T	P value	95% CI
Age (years)	32.32 (12.06)	38.57 (11.73)	0.65 (69)	-2.21	≤ 0.05*	-11.88/-0.61
Age at disease onset (years)	15.21 (2.79)	27.83 (9.88)	19.57(40.8)	-7.24	≤ 0.01 ^†^	-15.88/-8.96
Duration of disease (years)	16.91(12.67)	10.83 (10.82)	2.19 (68)	2.16	≤ 0.05*	0.47/11.68
ETISR-SF subdomains						
General trauma	3.47 (2.56)	2.83 (2.55)	0.08 (66)	1.02	NS	-0.61/1.87
Physical abuse	2.13 (1.77)	1.25 (1.54)	2.98 (66)	2.17	≤ 0.05*	0.07/1.67
Emotional abuse	2.56 (1.90)	1.94 (1.90)	0.00 (66)	1.34	NS	-0.30/1.53
Sexual abuse	1.16 (1.97)	0.94 (1.62)	0.84 (66)	0.49	NS	-0.66/1.08
Reaction to trauma	0.97 (0.82)	0.61 (0.90)	0.15 (66)	1.70	NS	-0.06/0.78
Total score	10.28 (6.01)	7.14 (6.36)	0.06 (66)	2.08	≤ 0.05*	0.14/6.15
Calgary depression	5.89 (6.34)	4.18 (4.46)	3.60 (53)	1.16	NS	-1.24/4.66
Liebowitz subdomains						
Fear and anxiety	20.55 (18.18)	17.09 (15.65)	0.14 (66)	0.84	NS	-4.74-11.65
Avoidance	17.58 (18.72)	15.60 (15.28)	0.46 (66)		NS	-6.22/10.23
PANSS						
Positive symptoms	17.76 (8.45)	18.94 (7.83)	0.13 (67)		NS	-5.10/2.72
Negative symptoms	21.00 (12.24)	21.03 (7.35)	11.41(51.5)		NS	-4.96/4.90
Total score	78.55 (34.30)	76.94 (16.89)	12.86 (45.7)		NS	-11.69/14.89

AOP = adult-onset psychosis (> 18 years); Calgary = Calgary Scale for Depression in Schizophrenia; EOP = early-onset psychosis (≤ 18 years); ETISR-SF = Early Trauma Inventory Self Report-Short Form; Liebowitz = Liebowitz Social Anxiety Scale; NS = not significant; PANSS = Positive and Negative Syndrome Scale.

Significant differences at an α level of * p ≤ 0.05 and ^†^ p ≤ 0.01.

### ETISR-SF and age at disease onset analysis

For the entire sample ( Table 4 ), the ETISR-SF sexual abuse score was positively correlated with the duration (in years) of the disease (r = 0.327, *p* ≤ 0.01); ETISR-SF emotional abuse also correlated inversely with age at first psychotic episode (r = -0.249, *p* ≤ 0.05). Table 5 shows correlations between ETISR-SF scale results and the clinical characteristics of groups. More frequent report of sexual abuse was correlated with longer disease duration in the EOP and with earlier breakdown of psychosis in the AOP; more frequent report of physical abuse was associated with longer disease duration in the AOP.

**Table 4:** Clinical comparisons of the entire sample according to age at disease onset

	Age at first psychotic episode	Duration of disease	ETISR-SF						Calgary	Liebowitz		PANSS		

			General trauma	Physical abuse	Emotional abuse	Sexual abuse	Reaction to trauma	Total score	Total	Fear and anxiety	Avoidance	Positive symptoms	Negative symptoms	Total score
Age (years)	0.306*	0.646*	0.064	-0.019	-0.167	0.186	-0.156	-0.036	0.110	0.150	0.164	-0.190	-0.150	**-0.290** ^**†**^
Age at first psychotic episode		-0.391*	-0.141	-0.213	**-0.249** ^**†**^	-0.170	-0.234	-0.315*	-0.035	0.069	0.145	-0.069	0.071	0.043
Duration of disease			0.159	0.161	0.052	**0.327** *	-0.027	0.199	0.169	0.036	0.007	-0.101	-0.207	**-0.282** ^**†**^
ETISR-SF subdomains														
General trauma				0.542*	0.347*	0.295 ^**†**^	0.561*	0.763*	0.442*	0.136	0.070	-0.083	-0.168	-0.219
Physical abuse					0.550*	0.214	0.635*	0.759*	**0.299** ^**†**^	**0.275** ^**†**^	0.187	-0.109	-0.159	-0.206
Emotional abuse						0.352*	0.561*	0.731*	0.186	0.176	0.130	-0.055	-0.020	-0.087
Sexual abuse							0.226	0.489*	0.179	0.125	0.055	0.112	-0.129	-0.136
Reaction to trauma								0.689*	0.174	**0.320** *	0.217	-0.079	-0.107	-0.129
Total									0.373*	0.167	0.111	-0.061	-0.150	-0.231
Calgary (total)										**0.323** ^**†**^	**0.313** ^**†**^	0.064	0.076	0.164
Liebowitz														
Fear and anxiety											0.917*	-0.037	0.075	0.175
Avoidance												-0.050	**0.248** ^**†**^	**0.262** ^**†**^
PANSS														
Positive symptoms													0.198	0.644*
Negative symptoms														0.754*

Calgary = Calgary Scale for Depression in Schizophrenia; ETISR-SF = Early Trauma Inventory Self Report-Short Form; Liebowitz = Liebowitz Social Anxiety Scale; PANSS = Positive and Negative Syndrome Scale.

Significant differences at an α level of * p ≤ 0.01 (Pearson correlations) and ^**†**^ p ≤ 0.05.

**Table 5:** Correlations between ETISR-SF scale scores and sociodemographic and clinical characteristics, Calgary scores, and Liebowitz scores for the EOP and AOP groups

Variables	ETISR-SF domains					

	General trauma	Physical abuse	Emotional abuse	Sexual abuse	Reaction to trauma	Total
Age (years)						
EOP	0.069	-0.131	-0.222	0.398*	-0.172	0.033
AOP	0.100	0.201	-0.077	-0.076	-0.074	0.020
Age at disease onset (years)						
EOP	0.069	0.128	-0.069	-0.134	0.012	-0.015
AOP	-0.112	-0.174	-0.323	-0.402*	0.000	-0.240
Duration of disease (years)						
EOP	0.040	-0.158	-0.200	0.410*	-0.174	0.024
AOP	0.255	0.353*	0.188	0.255	-0.036	0.267
Calgary Scale (total)						
EOP	0.293	0.217	0.065	-0.067	0.089	0.224
AOP	0.555 ^†^	0.356	0.278	0.423	0.257	0.467*
Liebowitz Scale						
Fear and anxiety						
EOP	0.146	0.335	0.259	0.136	0.462 ^†^	0.305
AOP	0.128	0.247	0.098	0.156	0.171	0.033
Avoidance						
EOP	0.009	0.194	0.173	0.047	0.330	0.177
AOP	0.128	0.206	0.071	0.082	0.113	0.033

AOP = adult-onset psychosis (> 18 years); EOP = early-onset psychosis (≤ 18 years); ETISR-SF = Early Trauma Inventory Self Report-Short Form; PANSS = Positive and Negative Syndrome Scale.

Significant differences to an α level of * p ≤ 0.05 and ^†^ p ≤ 0.01.

### ETISR-SF and Calgary, Liebowitz and PANSS analysis for the entire sample

Total PANSS score was inversely correlated with age (r = -0.290, *p* ≤ 0.05) and duration of disease (r = -0.282, p ≤ 0.05); The Calgary scale showed a positive statistical correlation with the ETISR-SF subscales general trauma (r = 0.442; p < 0.01) and physical abuse (r = 0.299; p < 0.05) and with total ETISR-SF score (r = 0.373; p < 0.01).

The fear/anxiety category of the Liebowitz Social Anxiety Scale also showed a positive correlation with physical abuse (r = 0.275; p<0.05) and reaction to trauma (r = 0.319; p < 0.01).

### ETISR-SF and Calgary, Liebowitz, and PANSS analysis for EOP and AOP subsets

Correlations for the EOP and AOP groups can be found in Table 4 . The item fear and anxiety from the Liebowitz scale shows a positive correlation with ETISR-SF reaction to trauma in the EOP group ( Table 4 ). Positive symptoms on the PANSS were correlated with younger disease onset in both the EOP (r = -0.364; p < 0.05) and AOP groups (r = -0.365; p < 0.05). Conversely, negative symptoms on the PANSS were negatively correlated with disease duration in the EOP group (r = -0.499; p < 0.01) and positively with age in the AOP group (r = 0.351; p < 0.05). No significant correlations were found between positive or negative PANSS symptoms and the ETISR-SF subdomains in either the EOP or the AOP groups.

### Hierarchical regression models

In the hierarchical regression, only two models were identified as potential predictors of PANSS scores ( Table 6 ). Of these, the model that achieved better prediction for the PANSS score was the one combining duration of disease and age of first psychotic episode, although with only 6.3% of the variance ( Table 6 ).

**Table 6:** Sum of model (hierarchical regression)

Model	R	R square	Adjusted R square	Standard error estimate	Statistics of change				

					Change in R Square	F	df1	df2	Sig. F change
1	0.303 ^a^	0.092	0.063	24.069	0.092	3.190	2	63	0.048
2	0.265 ^b^	0.070	0.056	24.162	-0.022	1.496	1	63	0.226
Dependent variable: PANSS total score.									
^a^ Predictors (1) = duration of disease and age of first psychotic episode.									
^b^ Predictors (2) = duration of disease.									

**Model**		**Nonstandard coefficients**		**Standard coefficients**	**t**	**Sig.**			

		**B**	**Standard error**	**Beta**					

1	(Constant)	94.745	9.958		9.515	0.000			
	Age at first psychotic episode	-0.405	0.331	-0.160	-1.223	0.226			
	Duration of disease	-0.674	0.268	-0.330	-2.515	0.014			
2	(Constant)	83.941	4.617		18.182	0.000			
	Duration of disease	-0.543	0.247	-0.265	-2.202	0.031			

**Excluded variables**									

**Model**	**ETISR-SF subdomains**	**Beta In**	**T**	**Sig.**	**Partial correlation**	**Collinearity statistics (tolerance)**			

1	General trauma	-0.165 ^b^	-1.354	0.181	-0.170	0.962			
	Physical abuse	-0.100 ^b^	-0.812	0.420	-0.103	0.962			
	Emotional abuse	-0.040 ^b^	-0.321	0.749	-0.041	0.948			
	Sexual abuse	-0.077 ^b^	-0.586	0.560	-0.074	0.845			
	Reaction to trauma	-0.083 ^b^	-0.680	0.499	-0.086	0.975			
	Total score	-0.146 ^b^	-1.181	0.242	-0.148	0.933			
2	General trauma	-0.161 ^c^	-1.322	0.191	-0.164	0.963			
	Physical abuse	-0.082 ^c^	-0.668	0.507	-0.084	0.974			
	Emotional abuse	-0.005 ^c^	-0.040	0.968	-0.005	0.999			
	Sexual abuse	-0.060 ^c^	-0.458	0.649	-0.058	0.853			
	Reaction to trauma	-0.060 ^c^	-0.490	0.626	-0.062	0.997			
	Total score	-0.120 ^c^	-0.970	0.336	-0.121	0.956			
	Age of first psychotic episode	-0.160 ^c^	-1.223	0.226	-0.152	0.839			

Dependent variable = PANSS total score.

Predictors (1) = duration of disease and age of first psychotic episode.

Predictors (2) = duration of disease.

ETISR-SF = Early Trauma Inventory Self Report-Short Form; PANSS = Positive and Negative Syndrome Scale.

## Discussion

**Figure 1:**
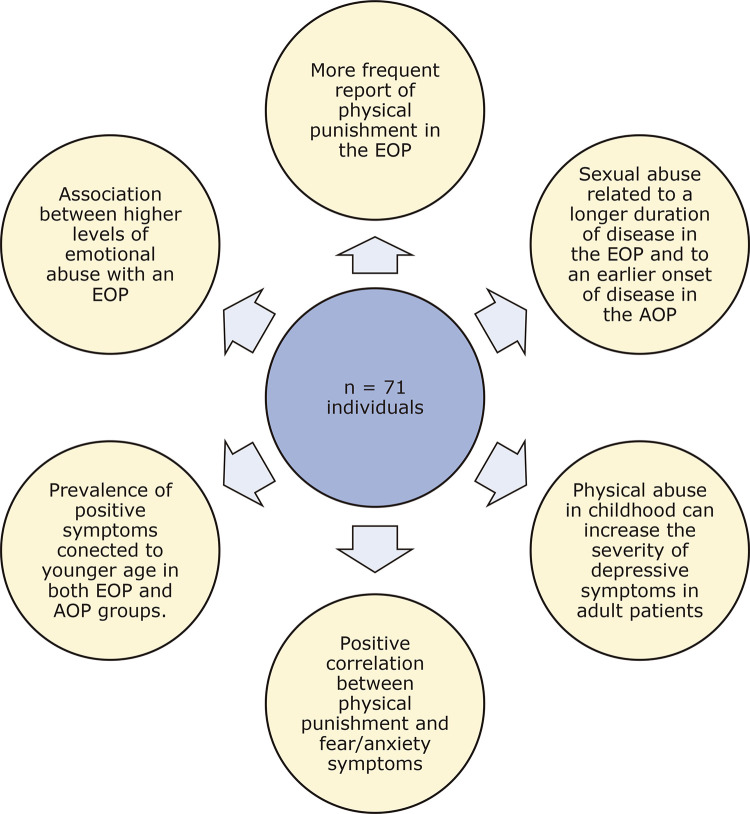
Summary of the main findings. AOP = adult onset psychosis; EOP = early onset psychosis.

The current study investigated the prevalence of early trauma in schizophrenia and its relationships with clinical symptoms and with the age of onset of the disease and highlighted significant findings: first, there was an association between higher levels of trauma in the ETISR-SF, particularly the emotional abuse dimension, and earlier onset of schizophrenia; also, history of sexual abuse was associated with longer duration of disease in the EOP and with earlier onset of the disease in the AOP; finally, the prevalence of positive symptoms was associated with younger age in both early and adult-onset groups ( Figure 1 ).

The most important finding was a more frequent report of physical abuse among EOP subjects. Our results are in line with findings showing an association between traumatic experiences such as physical violence and the onset of psychosis at an earlier age. ^8 , 15 , 16 , 29^ In a recent population cohort with 4433 patients with an average age of 17.8 years, exposure to any trauma up to 17 years of age was associated with increased psychotic experiences at the age of 18 (adjusted odds ratio, 2.91; 95%CI, 2.15-3.93). All types of trauma from early childhood to late adolescence were associated with an increased chance for psychotic experiences. The fraction attributable to the population for childhood and adolescence trauma in psychotic experiences at 18 was 45% (95%CI, 25% -60%). ^29^

### Depression and traumata in SZ

Another association worth mentioning was found between the Calgary Depression Scale and the ETISR-SF total score and general traumata and physical punishment subdomains, suggesting that physical maltreatment in childhood may increase the severity of depressive symptoms in adult patients with schizophrenia. This is in line with previous findings, for instance, Gilbert et al., ^30^ which shows the relationship between child maltreatment and increased risk of depression and anxious behavior in adulthood and aggressive and violent behavior. In this sense, children who are victims of maltreatment have a moderately increased risk of depression in adolescence and adulthood, only partially reflecting the family context in which abuses occur. ^31^ About one-quarter to one-third of children who are victims of maltreatment meet the DSM IV criteria for major depression by the age of 20. ^16^ Our findings constitute evidence that depressive symptoms are associated with physical abuse and general traumata, with no specific effect of any maltreatment being found. Some researchers, however, found evidence of response to the severity of the abuse. Diagnosis of depression could be more closely related to severe physical abuse than to less severe maltreatment forms. ^16 , 32^ Also, early exposure to trauma and stress predicted higher depression scores, also measured by the dysthymia category on the PANSS scale. ^8^ For many affected individuals, the onset of depression begins in childhood, reinforcing the need for early intervention in the lives of abused and neglected children before symptoms of depression influence other domains of functioning. ^33^

### Social anxiety, traumata, and SZ

The association between the fear/anxiety category on the Liebowitz Social Anxiety Scale and reaction to trauma in the EOP also merits being highlighted as a relevant finding. There is still little evidence on the effect of childhood trauma exposure and social anxiety among high-risk schizophrenia patients. One meta-analysis conducted in non-schizophrenia individuals with social anxiety reported a high prevalence of other early traumata (median: 18% to 45%). ^34^ Results indicated that early exposure to trauma was 1.9 to 3.6 times more likely to result in anxiety disorders in comparison with healthy individuals. ^34^ Contrasting with our findings, the emotional trauma dimension was the most important risk factor for social anxiety disorder. Conversely, no associations were found between avoidance symptoms on the Liebowitz Scale and any of the ETISR-SF subdomains. These findings contrast with recent evidence from Gabínio et al., ^10^ who reported that children with an early unstable emotional foundation are more vulnerable and reactive to stressful situations and to development of psychopathological changes over time. In this sense, individuals with anxiety would develop hypersensitivity to failures and frustrations resulting from interpersonal relationships, reacting with avoidant behaviors, which would be reflected in relationships established throughout their lives.

### Positive-negative symptoms and traumata

Regarding the prevalence of positive-negative symptoms, our study observed some results worth mentioning for the EOP group. First, an association between positive symptoms and younger disease breakdown; second, negative symptoms correlated with disease duration in this group. Finally, in the logistic regression model, younger disease onset combined with longer duration also predicted positive-negative symptoms. Whether exposure to early trauma may be related to the development of negative-positive symptoms is largely unknown and remains disputable. In a review conducted by Ruby et al., ^8^ there was a paucity of studies investigating the putative relationship between negative symptoms and childhood abuse. Although somewhat controversial, the results reported here are supported by previous studies. ^35 , 36^ In one such study, investigating paranoid schizophrenia or other psychotic related disorders, childhood trauma was significantly and positively associated with negative symptoms, post-traumatic stress disorders, and dissociation. ^35^ Additionally, a longitudinal study with young individuals with high clinical risk for schizophrenia found that impaired tolerance of stress was related to negative symptoms over time, putatively via an increased sensitivity to stress. ^36^ However, negative results have also been described by some studies. Absence of any relation between early trauma and negative symptoms in psychotic subjects, including individuals in the first psychotic episode, and in adolescents and young adults with clinical high-risk for psychosis has been reported. ^37 - 39^

### Putative mechanisms of traumata and risk of SZ

Hypothetical mechanisms linking emotional and social experiences with early neurobiological abnormalities in SZ have been intensely debated. ^33 , 36 - 38^ Possibly, early life experiences, combined with genetic factors, exert a significant influence on behavioral patterns presented in adulthood. In childhood and adolescence, functional and connectivity changes may occur in several brain regions as part of the maturation process. Hence, stressful events experienced early in life may influence brain development, as demonstrated by neuroimaging and neuropsychological studies. ^40^ These findings point to a study that showcased significant associations between early traumata and higher levels of experiential anomalies, which can be considered disturbances of self-awareness in patients in the early stages of schizophrenia. In turn, self-awareness disturbances seem to be triggered by emotional abuse, emotional neglect, and physical neglect, in addition to the frequent relation between sexual and physical abuse and development of psychotic features, as well as prodromal symptomatology. ^41^ Previous literature has demonstrated the correlation between diagnosis of schizophrenia and early neglect, while non-psychotic disorders were correlated to various abuses. ^35^ Taken as a whole, our findings highlight the relevance of traumatic childhood experiences to the development of a primary psychotic disorder in biologically vulnerable individuals.

### Limitations

This study has some significant limitations that deserve further comment. Firstly, the limited sample, all deriving from one psychiatric service in the city of São Luís, northern Brazil. Due to these aspects, results may be influenced by ecological aspects and reduced statistical power and should be regarded as preliminary. The reduced statistical power may also explain the small proportion of variance in PANSS symptoms explained by the predictors. Another caveat is the retrospective method of assessment of traumatic experiences, which may introduce selection bias and misclassification or information bias. Furthermore, the temporal relationship between early trauma exposure and psychosis is frequently difficult to assess. The research project has already enrolled a larger number of patients and future analyses will clarify some of the issues mentioned above and confirm our hypotheses.

## Conclusions

Our study investigated emotional and physical traumata in subjects with schizophrenia. The results support the hypothesis that early exposure to trauma may play an important role in developing psychotic symptoms in adulthood, favoring an earlier disease onset and, possibly, other psychiatric disorders, such as fear and anxiety in the context of social anxiety and depression. Although preliminary, our findings support the hypothesis linking socio-environmental experiences in childhood and adolescence to development of schizophrenia at younger ages and emphasize the need for early therapeutic interventions in children and adolescents undergoing psychological stress, to prevent and reduce severe clinical features related to psychosis in adulthood. Finally, future work with larger samples and longitudinal projects will confirm our preliminary findings.
